# TaxAss: Leveraging a Custom Freshwater Database Achieves Fine-Scale Taxonomic Resolution

**DOI:** 10.1128/mSphere.00327-18

**Published:** 2018-09-05

**Authors:** Robin R. Rohwer, Joshua J. Hamilton, Ryan J. Newton, Katherine D. McMahon

**Affiliations:** a Environmental Chemistry and Technology Program, University of Wisconsin—Madison, Madison, Wisconsin, USA; b Department of Bacteriology, University of Wisconsin—Madison, Madison, Wisconsin, USA; c School of Freshwater Sciences, University of Wisconsin—Milwaukee, Milwaukee, Wisconsin, USA; d Department of Civil and Environmental Engineering, University of Wisconsin—Madison, Madison, Wisconsin, USA; University of California, Davis

**Keywords:** 16S rRNA gene, amplicon sequencing, freshwater, limnology, microbial ecology, taxonomy, taxonomy assignment, taxonomy database

## Abstract

Taxonomy assignment of freshwater microbial communities is limited by the minimally curated phylogenies used for large taxonomy databases. Here we introduce TaxAss, a taxonomy assignment workflow that classifies 16S rRNA gene amplicon data using two taxonomy reference databases: a large comprehensive database and a small ecosystem-specific database rigorously curated by scientists within a field. We applied TaxAss to five different freshwater data sets using the comprehensive SILVA database and the freshwater-specific FreshTrain database. TaxAss increased the percentage of the data set classified compared to using only SILVA, especially at fine-resolution family to species taxon levels, while across the freshwater test data sets classifications increased by as much as 11 to 40% of total reads. A similar increase in classifications was not observed in a control mouse gut data set, which was not expected to contain freshwater bacteria. TaxAss also maintained taxonomic richness compared to using only the FreshTrain across all taxon levels from phylum to species. Without TaxAss, most organisms not represented in the FreshTrain were unclassified, but at fine taxon levels, incorrect classifications became significant. We validated TaxAss using simulated amplicon data derived from full-length clone libraries and found that 96 to 99% of test sequences were correctly classified at fine resolution. TaxAss splits a data set’s sequences into two groups based on their percent identity to reference sequences in the ecosystem-specific database. Sequences with high similarity to sequences in the ecosystem-specific database are classified using that database, and the others are classified using the comprehensive database. TaxAss is free and open source and is available at https://www.github.com/McMahonLab/TaxAss.

**IMPORTANCE** Microbial communities drive ecosystem processes, but microbial community composition analyses using 16S rRNA gene amplicon data sets are limited by the lack of fine-resolution taxonomy classifications. Coarse taxonomic groupings at the phylum, class, and order levels lump ecologically distinct organisms together. To avoid this, many researchers define operational taxonomic units (OTUs) based on clustered sequences, sequence variants, or unique sequences. These fine-resolution groupings are more ecologically relevant, but OTU definitions are data set dependent and cannot be compared between data sets. Microbial ecologists studying freshwater have curated a small, ecosystem-specific taxonomy database to provide consistent and up-to-date terminology. We created TaxAss, a workflow that leverages this database to assign taxonomy. We found that TaxAss improves fine-resolution taxonomic classifications (family, genus, and species). Fine taxonomic groupings are more ecologically relevant, so they provide an alternative to OTU-based analyses that is consistent and comparable between data sets.

## Abstract (precis)

Microbial communities drive ecosystem processes, but microbial community composition analyses using 16S rRNA gene amplicon data sets are limited by the lack of fine-resolution taxonomy classifications. Coarse taxonomic groupings at the phylum, class, and order levels lump ecologically distinct organisms together. To avoid this, many researchers define operational taxonomic units (OTUs) based on clustered sequences, sequence variants, or unique sequences. These fine-resolution groupings are more ecologically relevant, but OTU definitions are data set dependent and cannot be compared between data sets. Microbial ecologists studying freshwater have curated a small, ecosystem-specific taxonomy database to provide consistent and up-to-date terminology. We created TaxAss, a workflow that leverages this database to assign taxonomy. We found that TaxAss improves fine-resolution taxonomic classifications (family, genus, and species). Fine taxonomic groupings are more ecologically relevant, so they provide an alternative to OTU-based analyses that is consistent and comparable between data sets.

## INTRODUCTION

Microbial communities form the foundations of all ecosystems, yet interpretation of community data is limited by the difficulty of comparison across data sets. With the rapid development of massively parallel sequencing technology, scientists are increasingly able to fingerprint microbial communities using amplicon sequencing of marker genes such as the 16S rRNA gene. The resulting sequences are typically grouped into operational taxonomic units (OTUs) defined by sequence identity or sequence variants. Comparison between amplicon data sets is difficult because OTUs are specific to each analysis. For clarity, this article refers to 16S rRNA gene amplicon sequencing data sets as “data sets” and defines OTUs as a data set’s sequence unit of measure, irrespective of whether those units represent clustered sequences, sequence variants, or unique sequences.

### Taxonomy allows cross-study analyses.

OTUs are widely used to represent ecologically coherent entities (1); however, they represent study-specific phylotypes that cannot be compared between data sets. Many common OTU definitions, including sequence identity-based clustering (2), minimum entropy decomposition (3), and distribution-based clustering (4), are specific to each analysis, resulting in arbitrary OTU names. OTU definitions based on exact sequences, such as DADA2’s denoising approach (5) or defining OTUs as unique sequences, are still specific to the amplicon region and sequencing platform used in each study. For these reasons, direct comparison of OTUs between multiple data sets is most often impossible.

Taxonomic naming systems allow comparisons between data sets by creating consistent terminology and consistent phylogeny-determined boundaries between organisms. However, taxonomic naming is most useful when sequences can be classified to a fine level (e.g., family, genus, or species). Many abundant taxa have poorly resolved fine-scale phylogenetic structures in reference taxonomy databases (here “databases”), resulting in only coarse classifications for large proportions of amplicon data sets (e.g., phylum, class, or order). Coarse taxonomic groupings often include diverse organisms with different ecological roles, so analyses at coarse taxon levels miss underlying ecological dynamics (6). Fine-resolution taxonomic names are required to bridge the gap between ecologically relevant OTU-based analyses and consistent, comparable taxonomy-based analyses.

### Ecosystem-specific taxonomy databases.

Microbial ecologists from diverse subfields have created fine-resolution reference taxonomies by curating databases specific to their ecosystems. These ecosystem-specific databases are small compared to the large comprehensive databases compiled by Greengenes (7), SILVA (8), and the Ribosomal Database Project (9), but they are generally well curated with more finely resolved phylogenies for ecosystem-specific lineages. Examples of ecosystems with curated databases include the human oral cavity (10), the cow rumen (11), the honey bee gut (12), the cockroach and termite gut (13), activated sludge (14), and freshwater lakes (15). Ecosystem-specific databases are created to establish consistent vocabulary for common uncultured bacteria, create monophyletic reference structures, incorporate new reference information, and understand what the “typical” organisms are in a given ecosystem. Additionally, ecosystem-specific databases can be used to assign taxonomy to a finer resolution than can be achieved with a large comprehensive database.

### The FreshTrain.

This paper demonstrates TaxAss’s efficacy using a variety of freshwater amplicon data sets, the comprehensive SILVA database (8), and the ecosystem-specific Freshwater Training Set (FreshTrain) (15). The FreshTrain database was created in 2012 and was originally curated alongside Greengenes. FreshTrain versions match Greengenes and SILVA at the phylum, class, and order levels, but at finer taxonomic levels, the FreshTrain is curated based on additional information, such as the geographical distribution of sequences. These finer levels are referred to as lineage, clade, and tribe and approximate the Linnaean family, genus, and species (15). The FreshTrain is available online at https://www.github.com/McMahonLab/TaxAss.

### Taxonomy assignment algorithm.

Classification algorithms assign taxonomic names to OTUs based on their similarity to reference sequences in a database. The most commonly used classification algorithm was developed by Wang et al. (16) for the Ribosomal Database Project and is implemented in both mothur (17) and QIIME (18). This naive Bayesian classifier (here the “Wang classifier”) assigns taxonomy to OTUs based on 8-mer signatures and reports a bootstrap confidence estimate for each assignment (16). This bootstrap confidence value is based on the repeatability of the OTU’s assignment with subsampled 8-mers, not on an absolute similarity measure. In a large database, an OTU dissimilar to any reference sequences will not be classified repeatably as any one taxon, resulting in a low bootstrap confidence. However, in a small database, an OTU dissimilar to any reference sequences nevertheless can be classified repeatably because there are fewer references from which to choose. We refer to this pitfall as “misclassification” when OTUs are classified as unrelated organisms and “overclassification” when OTUs are classified to a finer taxon level than warranted.

### Introducing TaxAss.

We aimed to obtain fine-level taxonomy classifications in freshwater data sets by leveraging the ecosystem-specific FreshTrain database, while at the same time maintaining the full biological diversity of each data set. To this end, we developed an open source taxonomy assignment workflow (TaxAss) that uses the popular Wang classifier as implemented in mothur and employs both an ecosystem-specific database and a comprehensive database. TaxAss maintains taxonomic richness and accuracy by only classifying OTUs that share a high percent identity with ecosystem-specific reference sequences using the ecosystem-specific database. The remaining OTUs are classified using the comprehensive database. TaxAss scripts and step-by-step directions are available online at https://www.github.com/McMahonLab/TaxAss.

## RESULTS

### Methods summary.

**FIG 1:**
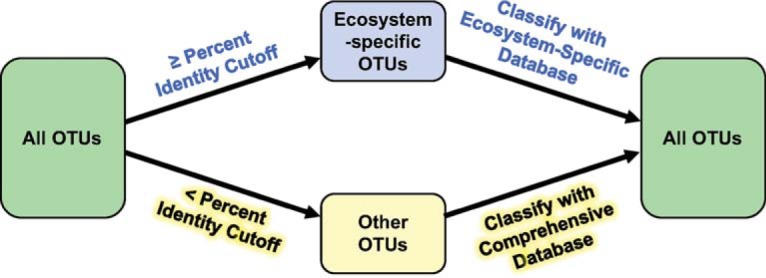
TaxAss conceptual diagram. TaxAss separates OTUs into two groups that are classified separately and then recombined. OTUs similar to any ecosystem-specific reference sequences are classified using the ecosystem-specific database; otherwise, they are classified by the comprehensive database. BLAST is used to split the OTUs into groups (left arrows), and the Wang classifier is used to assign taxonomy (right arrows).

TaxAss uses both an ecosystem-specific database and a large comprehensive database to improve taxonomic assignment resolution while maintaining richness. To classify the maximum possible number of OTUs and avoid misclassifications, overclassifications, and underclassifications, the amplicon data set is split into two groups using blastn prior to classification: OTUs with a high percent identity to ecosystem-specific reference sequences and OTUs with a low percent identity to ecosystem-specific reference sequences. The two groups are then classified separately using the Wang classifier and the appropriate database (Fig. 1).

To test TaxAss, we used SILVA version 132 and the FreshTrain as the comprehensive and freshwater-specific databases. We classified six 16S rRNA gene amplicon (“tag”) data sets spanning five freshwater ecosystems and a nonfreshwater control. Sequences in these tag data sets are not directly comparable because they cover five different amplicon regions. We defined OTUs as the unique sequences remaining after basic quality filtering and chimera checking.

### Assignment accuracy.

**FIG 2:**
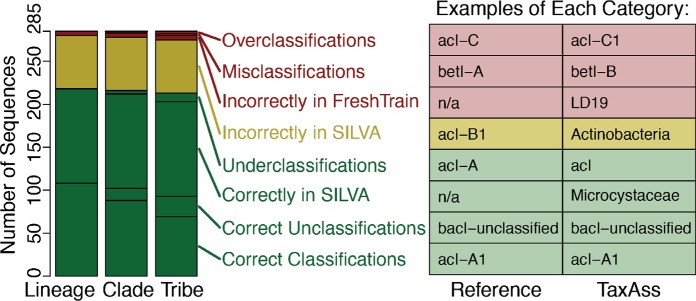
TaxAss validation with tags simulated from full-length Marathonas Reservoir clone libraries. Tags simulated by trimming full-length sequences to the V4 region were classified by TaxAss, and the resulting classifications were compared to “reference” classifications determined by manually aligning the full-length sequences to the FreshTrain. Correct classifications are in green, lost ecosystem-specific classifications are in yellow, and incorrect classifications are in red. (Left**)** Number of unique sequences in each classification category at fine-resolution taxon levels. (Right) Examples of classifications that fit into each classification category. Tabular results from this and additional amplicon region simulations are available in Table S1.

To test the accuracy of TaxAss’s taxonomy assignments, we compared TaxAss results to the gold standard provided by manual alignment of full-length 16S rRNA gene sequences. For this test, we used a full-length freshwater clone library data set from Marathonas Reservoir, Greece (19), which was not previously incorporated into the FreshTrain. We manually aligned these full-length sequences to the FreshTrain and then simulated a tag data set by trimming the full-length sequences to the commonly used primer regions V4, V4-V5, and V3-V4. We classified this simulated tag data set using TaxAss with the FreshTrain and SILVA and compared the results to the gold standard results provided by manual full-length alignments and phylogenetic analysis (Fig. 2).

We found that the majority (74.7%) of V4 tag sequences were classified correctly at the species/tribe level and that 86% of the incorrect assignments were due to sequences being classified using SILVA when they should have received FreshTrain nomenclature, which results in correct, though not ecosystem-specific, classifications. The remaining incorrect assignments stemmed from overclassification errors (1.1%), misclassification errors (0.7%), or incorrect inclusion in the FreshTrain classification set (1.8%), which can result in overclassification, misclassification, or underclassification (Fig. 2, left panel). Examples of each classification category are shown in the table in the right panel of Fig. 2. We do not consider underclassifications to be an error because underclassifications are expected due to the lower phylogenetic resolution of short tag sequences compared to full-length sequences. We found slightly lower error rates for the longer V4-V5 and V3-V4 amplicon regions (see Table S1 in the supplemental material).

10.1128/mSphere.00327-18.7 TABLE S1  Simulated V4, V4-V5, and V3-V4 tag classification accuracy as determined by comparison to their full-length alignment-based taxonomies. Download TABLE S1, PDF file, 0.05 MB. Copyright © 2018 Rohwer et al. 2018 Rohwer et al. This content is distributed under the terms of the Creative Commons Attribution 4.0 International license.

### Fine-resolution classifications increased.

**FIG 3:**
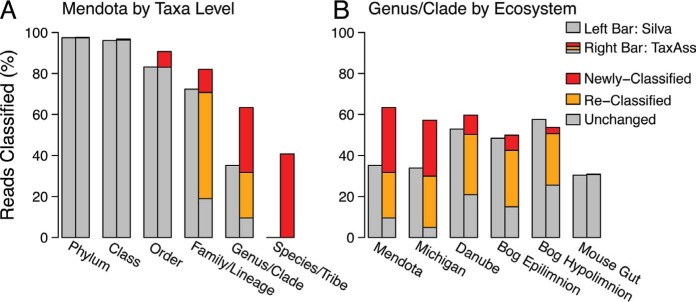
TaxAss performance compared to SILVA-only performance. (A and B) The left bars represent the SILVA-only classification, and the right bars represent the TaxAss classification that leveraged both SILVA and the FreshTrain. Within the right bars, red reads were classified by the FreshTrain using TaxAss and were unclassified using only SILVA; yellow reads were classified by the FreshTrain using TaxAss but received SILVA classifications using only SILVA, and gray reads remained classified by SILVA when using TaxAss. (A) In the Lake Mendota data set, TaxAss leveraged the FreshTrain and SILVA to achieve improved fine-resolution classifications. (B) TaxAss achieved improvements in a range of freshwater data sets despite the FreshTrain’s primary focus on temperate lake epilimnia. Few changes in classification were observed in the mouse gut control. Versions of this figure across all data sets and taxa levels can be found in Fig. S1.

To test whether TaxAss improved taxonomic classification over solely using a comprehensive database, we assigned taxonomy to a Lake Mendota amplicon data set first by using SILVA alone and then by using TaxAss to leverage both SILVA and the FreshTrain (Fig. 3A; Table 1). We compared the percentages of reads classified by both methods and observed a marked improvement in the percentage of the data set classified to the fine taxon levels of family/lineage, genus/clade, and species/tribe. At the species/tribe level, the percentages of reads classified increased from 0% to 41%, at the genus/clade level they increased from 35% to 63%, and at the family/lineage level they increased from 72% to 82%. In addition to these increases in classifications, TaxAss also improved the quality of classifications because the FreshTrain is curated with terminology and phylogeny consistent with the freshwater microbial ecology literature. For example, the abundant and cosmopolitan freshwater tribe acI-A1 is split into the “hgcl clade” and “*Ca*. Planktophila” in SILVA, acI-A4 and -A5 are also grouped with SILVA’s “hgcl clade,” and acI-A3 is grouped with “*Ca*. Planktophila.” At the family/lineage level, SILVA alone could classify a majority of the data set, but 72% of those SILVA-classified reads received more meaningful ecosystem-specific nomenclature when using TaxAss.

**TABLE 1:** Classification of the Lake Mendota data set^a^ using SILVA alone, the FreshTrain alone, or TaxAss to leverage both databases

Taxonomy level	% classified by^b^:			Taxonomic richness with^c^:		
	SILVA	TaxAss	FreshTrain	SILVA	TaxAss	FreshTrain
Phylum	97	98	85	63	63	6
Class	96	97	84	160	162	10
Order	83	91	76	387	388	31
Family/lineage	72	82	69	700	742	57
Genus/clade	35	63	56	1,468	1,529	94
Species/tribe	0	41	41	1,468	1,579	147

^a^ Versions of this table for each tested data set can be found in Table S2.

^b^ % classified = total reads classified/total reads in data set × 100%.

^c^ Taxonomic richness represents total unique classifications.

The FreshTrain reference sequences come exclusively from temperate lake epilimnia, and many of them come from Lake Mendota itself. Lake Mendota is a eutrophic, temperate lake in Wisconsin, and the Lake Mendota amplicon data set consists of 95 epilimnetic samples collected by the North Temperate Lakes Microbial Observatory over 11 years. To test TaxAss’s efficacy when the ecosystem-specific database is less representative of the ecosystem under investigation, we classified amplicon data sets from a range of freshwater ecosystems, first by using SILVA alone and then by using TaxAss to leverage SILVA and FreshTrain (Fig. 3B). The additional ecosystems we chose included the epilimnion of oligotrophic Lake Michigan (20), the eutrophic Danube River (21), and the epilimnion and hypolimnion of dystrophic Trout Bog (WI) (22). We also used a mouse gut data set (23) as a negative control to ensure that TaxAss would not assign FreshTrain classifications erroneously. All freshwater data sets showed improvements at all fine taxon levels (Fig. 3B; see Fig. S1 in the supplemental material), with the amount of improvement reflecting the similarity of each ecosystem to the FreshTrain reference sequences. For example, the temperate Lake Mendota and Lake Michigan epilimnia received the most FreshTrain classifications (54 and 52% of total reads at the genus/clade level), while the dystrophic bog hypolimnion benefited least (28% at the genus/clade level). Only 0.1% of the mouse gut control data set received FreshTrain classifications at the species, genus, or family levels.

10.1128/mSphere.00327-18.4 FIG S1  TaxAss compared to SILVA-only performance. (A) Shown is a version of Fig. 3a for each data set used in this article. (B) Shown is a version of Fig. 3b for each taxon level. Download FIG S1, PDF file, 0.2 MB. Copyright © 2018 Rohwer et al. 2018 Rohwer et al. This content is distributed under the terms of the Creative Commons Attribution 4.0 International license.

### Richness maintained.

**FIG 4:**
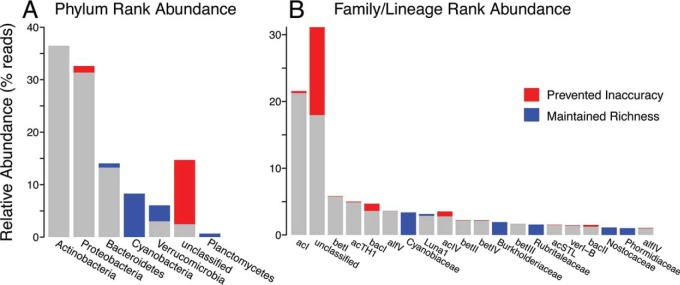
TaxAss performance compared to FreshTrain-only performance. (A and B) Lake Mendota reads represented by blue bars were incorrectly classified as red bars in the FreshTrain-only classification. Rank order of the bars follows the TaxAss classification rank abundances. Only taxa with at least 0.5% relative abundance are included, and at the lineage level, the number of bars displayed is further truncated to 20. (A) TaxAss maintained phylum richness (blue bars) by classifying phyla using SILVA when they are not included in FreshTrain. (B) TaxAss prevented lineage-level inaccuracies from misclassifications and overclassifications (red bars over known taxa) and lineage-level underclassifications (red bar over “unclassified” category). Versions of this figure across all test ecosystems can be found in Fig. S2.

To test whether TaxAss improved taxonomic classification over solely using an ecosystem-specific database, we assigned taxonomy to the Lake Mendota data set first by using the FreshTrain alone and then by using TaxAss to leverage both the FreshTrain and SILVA. TaxAss maintained taxonomic richness at all taxon levels by classifying OTUs into a larger variety of taxonomic names (Fig. 4; Table 1). At the same time, TaxAss prevented overclassifications and misclassifications at fine-resolution taxa levels compared to FreshTrain-only classifications (Fig. 4B).

10.1128/mSphere.00327-18.5 FIG S2  TaxAss performance compared to FreshTrain-only performance. Shown is a version of Fig. 4 for each data set used in this article. Download FIG S2, PDF file, 0.1 MB. Copyright © 2018 Rohwer et al. 2018 Rohwer et al. This content is distributed under the terms of the Creative Commons Attribution 4.0 International license.

The FreshTrain is a more specific database with less taxonomic richness than SILVA, so a decrease in taxonomic richness in a FreshTrain-only classification was expected. For example, the FreshTrain focuses on heterotrophic bacteria and does not include any *Cyanobacteria*, which comprised 8.3% of the Lake Mendota data set. All of Lake Mendota’s cyanobacterial OTUs were classified as something else (99.9% as unclassified), which resulted in a loss of phylum-level richness in the FreshTrain-only classification (Fig. 4A). In contrast, TaxAss maintained the taxonomic richness of a SILVA-only classification (Table 1; see Table S2 in the supplemental material).

10.1128/mSphere.00327-18.8 TABLE S2  Classification of all tested data sets using SILVA alone, the FreshTrain alone, or using TaxAss to leverage both databases. Shown is a version of Table 1 for each data set used in this article. Footnote *a*: % classified = total reads classified/total reads in data set × 100%. Footnote *b*: taxonomic richness represents total unique classifications. Download TABLE S2, PDF file, 0.03 MB. Copyright © 2018 Rohwer et al. 2018 Rohwer et al. This content is distributed under the terms of the Creative Commons Attribution 4.0 International license.

We also observed that some OTUs that TaxAss classified using SILVA were misclassified or overclassified by the FreshTrain-only approach (Fig. 4B). These incorrect classifications by the small FreshTrain database were less common than underclassification errors, but they had significant effects on taxon relative abundances at finer-resolution taxon levels. Lake Mendota’s fifth most abundant lineage, *Bacteroidetes* bacI, gained 30% more reads in a FreshTrain-only classification compared to using TaxAss. The classification errors TaxAss prevented were significant enough to change basic attributes such as rank abundances of top taxa and had an even larger impact on the freshwater test data sets that differed more from the FreshTrain references (see Fig. S2 in the supplemental material).

### Percent identity cutoff.

**FIG 5:**
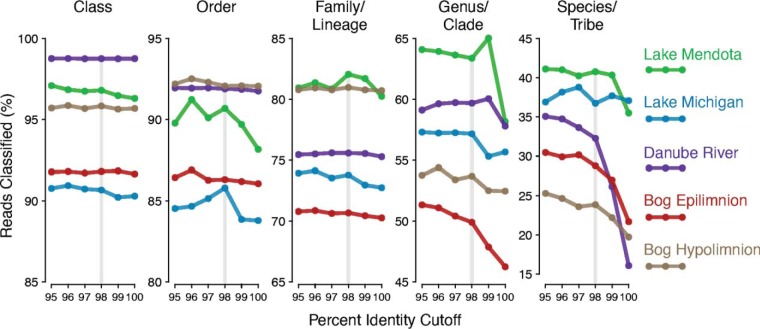
Percent identity where classifications are maximized. The percentages of reads classified when using different percent identity cutoffs to separate out ecosystem-specific OTUs are shown for each freshwater data set across taxon levels. Faint vertical lines highlight the 98% identity chosen for the analyses in this paper. OTUs are predominantly unclassified at fine resolution if they are placed in the wrong classification group, so this visualization is generated by TaxAss to help users choose a percent identity cutoff appropriate for their data set.

An OTU is classified using the ecosystem-specific database only if it matches a sequence in that database with a percent identity above a threshold set by the user. Therefore, the percent identity cutoff choice for taxonomic classification is central to the proper functioning of TaxAss because it determines which OTUs are classified in each database (ecosystem specific versus comprehensive). If the percent identity cutoff is set too high, ecosystem-specific OTUs are passed to the comprehensive database for classification; while if it is set too low, non-ecosystem-specific OTUs are passed to the ecosystem-specific database for classification. In both scenarios, the majority of misplaced OTUs will be unclassified at fine taxon levels. TaxAss allows users to compare the percentage of reads classified using different percent identity cutoffs, the idea being that a percent identity that maximizes reads classified has minimized misplacement errors of the abundant OTUs (Fig. 5).

We found that a percent identity cutoff of 98 to 99% was appropriate for the analyzed freshwater data sets, and we applied a cutoff of 98% when processing all data used in this article (Fig. 5). TaxAss allows users to choose a cutoff specific to their data by generating the plots shown in Fig. 5, but users who wish to save computational time can simply choose a percent identity cutoff and only run the classification once.

### BLAST conversion.

The calculation of percent identity for use in database selection is based on the percent identity returned by the National Center for Biotechnology Information’s Basic Local Alignment Search Tool (BLAST) (24). The default megablast settings are appropriate for our application because they have been highly optimized to find short, highly similar alignments. However, BLAST finds areas of local similarity, and there is no way to require BLAST to align the entire length of a query OTU’s sequence. 16S rRNA gene amplicon sequences are highly similar, and differences in taxonomic classification can be based on even a single mismatch in the amplified region. Therefore, we recalculated the percent identities BLAST returned into “full-length” percent identities for the entire query OTU’s sequence length (see Text S1 in the supplemental material and the equation under “Percent identity recalculation” in Materials and Methods).

10.1128/mSphere.00327-18.1 TEXT S1  BLAST percent identity recalculation. This 3-page document defines the BLAST terminology, derives the equation to recalculate percent identity, and provides an example alignment and calculation. Download TEXT S1, PDF file, 0.1 MB. Copyright © 2018 Rohwer et al. 2018 Rohwer et al. This content is distributed under the terms of the Creative Commons Attribution 4.0 International license.

We found that recalculating percent identity was necessary to prevent dissimilar OTUs from inclusion in the ecosystem-specific classification. For example, the FreshTrain does not include any reference sequences from the major freshwater phylum *Cyanobacteria*, so no cyanobacterial OTUs have high true percent identities to any references in the FreshTrain. We found that the percent identity recalculation was necessary to prevent some cyanobacterial OTUs from meeting the percent identity cutoff due to the original BLAST percent identities being based on only a short aligned section of the OTU sequence (see Fig. S3 in the supplemental material).

10.1128/mSphere.00327-18.6 FIG S3  Importance of percent identity recalculation. The phylum *Cyanobacteria* exemplifies why the percent identity recalculation is necessary. The ecosystem-specific FreshTrain database contains no cyanobacterial references, so cyanobacterial reads serve as a control for something that should be classified in the comprehensive SILVA classification group. However, BLAST returned hits with high percent identities for cyanobacterial OTUs due to the inclusion of short, high-sequence-identity partial alignments (red bars). After the TaxAss percent identity recalculation, the cyanobacterial OTUs had lower percent identities, and none were included in the FreshTrain classification group (gray bars). Download FIG S3, PDF file, 0.01 MB. Copyright © 2018 Rohwer et al. 2018 Rohwer et al. This content is distributed under the terms of the Creative Commons Attribution 4.0 International license.

We also found that it was necessary to recalculate the percent identity from several BLAST alignments (“hits”) for each OTU because the best BLAST hit did not always have the highest recalculated percent identity. TaxAss examines the top five BLAST hits, recalculates the percent identity of each, and then uses the highest recalculated percent identity to determine if an OTU meets the cutoff. To ensure enough BLAST hits were examined to consistently arrive at the highest possible recalculated percent identity, we calculated the proportion of times each BLAST hit number had the highest recalculated percent identity. In the Lake Mendota amplicon data set, the first BLAST hit almost always also had the best recalculated score, and the contribution of additional BLAST hits was very low, especially when only “good” hits above a stringent percent identity cutoff were considered (Table 2). In the Lake Mendota data set at the chosen 98 percent identity cutoff, 99.68% of the best hits found by BLAST were also the best recalculated hits, and only 0.07% of BLAST’s fifth hits were used. TaxAss generates a version of Table 2 for users’ individual data sets, and if they observe more high-number BLAST hits contributing to the best recalculated hit, they can increase the number of BLAST results used for the calculation.

**TABLE 2:** Agreement between BLAST and recalculated percent identities

BLAST result	Recalculated percent identity cutoff applied^a^:					
	95	96	97	98	99	100
Hit 1	96.8^b^	97.8	99.0	99.68	99.90	100
Hit 2	1.4	1.0	0.5	0.18	0.07	0
Hit 3	0.5	0.3	0.1	0.04	0.01	0
Hit 4	0.7	0.4	0.1	0.03	0.01	0
Hit 5	0.7	0.5	0.2	0.07	0.01	0

^a^ Calculations were performed only on sequences above the listed recalculated percent identities.

^b^ For example, 96.8% of BLAST’s first hits also had the highest recalculated percent identity.

## DISCUSSION

### Ecosystem-specific databases.

The need for curated ecosystem-specific databases has been recognized by microbial ecologists studying many ecosystems. TaxAss was developed specifically to leverage the Freshwater Training Set (FreshTrain) (15), but it could be applied to custom databases curated for other ecosystems: the dictyopteran gut microbiota reference database (DictDb) (13), the rumen and intestinal methanogen database (RIM-DB) (11), the honey bee database (HBDB) (12), the microbial database for activated sludge (MiDAS) (14), and the human oral microbiome database (HOMD) (10). These databases were created by starting with a comprehensive database such as SILVA or Greengenes and then recurating the reference sequences from the study ecosystem, sometimes also incorporating new reference sequences. Often during curation, phylogenies were collapsed to be monophyletic and incorporate new organisms, and abundant but unnamed organisms were given placeholder names to allow for consistent terminology among researchers.

DictDB, HBDB, and MiDAS are fully integrated with modified versions of the entire SILVA database, so a workflow like TaxAss that leverages two databases is not needed because the single merged database can be used in one step for taxonomy assignment. However, fully integrated databases can be difficult to maintain over time because new versions of each database will diverge from each other, and TaxAss provides a means to circumvent this divergence. The FreshTrain is an example of this divergence in action. The FreshTrain was originally integrated into the Hugenholtz database that eventually became Greengenes, and Greengenes was last updated in May 2013. In addition, SILVA now contains more total references and has been updated as recently as December 2017, so some researchers prefer to use the more recently updated SILVA as their comprehensive database. Similarly, the FreshTrain has been updated almost annually since its creation as new full-length 16S rRNA gene sequences from freshwater ecosystems became available. TaxAss allows microbial ecologists to use the most up-to-date versions of their preferred databases without performing or waiting for reconciliation of each release.

Once an ecosystem-specific database has diverged from the comprehensive database, as occurred with the FreshTrain, leveraging the ecosystem-specific database for taxonomy assignment is no longer straightforward. Reintegrating the ecosystem-specific database into the comprehensive database is more involved than simply concatenating databases and removing duplicated references because conflicting phylogenetic structures must be resolved. Analysis of community amplicon data is a fairly routine part of many studies for which extensive phylogenetic curation would fall outside the scope. The FreshTrain has been used in a variety of ways since it diverged from the current version of Greengenes, and it is often difficult to discern the specifics from cursory sentences in a paper’s methods section. TaxAss provides a well-documented and rigorously tested workflow to leverage two conflicting databases without extensive curation.

### Current FreshTrain usage.

The simplest way the FreshTrain has been used to assign taxonomy to amplicon data sets is as part of a separate, complementary analysis. For example, in a study of the River Thames Basin (25), FreshTrain and Greengenes classifications were displayed side by side, and separate metrics such as diversity indices were calculated for each. However, the bulk of the taxonomic analyses were carried out at the coarse phylum level, despite most abundant OTUs having FreshTrain nomenclature. When the FreshTrain is used independently, the loss of richness in taxonomic classifications (Fig. 4 and Table 1) makes it difficult to use ecosystem-specific classifications for entire data set analyses. TaxAss provides ecosystem-specific classifications without loss of taxonomic richness, thus allowing for a single comprehensive analysis.

Another straightforward approach has been to classify amplicon data sets sequentially—first using the FreshTrain and then reclassifying the unclassified sequences using a comprehensive database. For example, in a study of Lake Erken, Sweden (26), OTUs were first classified with the FreshTrain, and then unclassified OTUs were reclassified using SILVA. While this approach allows for a single analysis, the initial classification of all sequences with the small FreshTrain database can cause overclassification and misclassification errors (Fig. 4B). TaxAss prevents this by splitting the OTUs into two groups prior to classification.

These classification errors when using the FreshTrain to classify all OTUs were observed in a study of cyanobacterial blooms in Yanga Lake, Australia (27), where the authors observed that cyanobacterial OTUs were misclassified as heterotrophic bacteria. To prevent this, Greengenes was used for an initial classification, and then only OTUs assigned to phyla included in the FreshTrain database were reclassified and renamed with confidently assigned FreshTrain nomenclature. This approach prevented the misclassification of Yanga Lake’s abundant cyanobacterial OTUs, but it would not prevent overclassification of OTUs that belong to phyla included in the FreshTrain (*Verrucomicrobia*, *Bacteroidetes*, *Proteobacteria*, and *Actinobacteria*). In freshwater data sets such as bogs, rivers, and lake hypolimnia, many organisms belonging to FreshTrain phyla differ significantly from the lake epilimnion references included in the FreshTrain. TaxAss prevents overclassification and misclassification of OTUs of any phyla.

Another way to avoid the overclassifications and misclassifications observed with the Wang classifier is to use BLAST-based taxonomy assignment algorithms that determine assignments based on sequence similarity. Since the BLAST algorithm calculates an absolute similarity instead of a relative one, a similarity cutoff prevents classifications to dissimilar sequences. BLAST was used to assign FreshTrain taxonomy to sequences from boreal lakes in Quebec, Canada (28). However, unlike the Wang classifier, BLAST only takes into account individual reference sequences and ignores their encompassing phylogenetic structure. The BLAST-based algorithm from Classification Resources for Environmental Sequence Tags (CREST) (29) addresses this by taking a lowest common ancestor approach. Each query OTU is classified to the finest taxon level that its top BLAST hits share. The CREST algorithm has also been used to assign FreshTrain taxonomy to sequences obtained from the Danube River in southeastern Europe (21). This approach avoided overclassifications and misclassifications, and incorporated phylogenetic information in the taxonomy assignments; however, it does not maintain diversity by also leveraging a comprehensive database. Additionally, the Wang classifier is more robust at coarser taxon levels and for shorter sequences (29), and it is implemented in common tools like mothur and QIIME. TaxAss allows users to leverage both ecosystem-specific and comprehensive databases using the highly trusted and conveniently implemented Wang classifier.

### Future TaxAss usage.

We recommend all microbial ecologists studying freshwater systems use the FreshTrain and TaxAss to classify their 16S rRNA gene amplicon data sets. This will result in a consistent, specific, and comparable vocabulary throughout the field and will improve classification for analysis of individual data sets. We also recommend that microbial ecologists with different ecosystem-specific databases consider TaxAss when their databases diverge from the most up-to-date comprehensive database and phylogenetic curation is outside the scope of their project.

We recommend microbial ecologists create ecosystem-specific databases if one does not already exist, since they provide improved analysis and enhanced collaboration for the entire field. Phylogenies must be created from full-length 16S rRNA gene sequences, which are currently not collected as routinely as short amplicon sequences. However, we believe the benefit of these databases as a reference for the field and to improve taxonomic classification of amplicon sequences justifies the effort to create them, especially since TaxAss allows their use without constant recuration. Additional full-length sequences to flesh out the existing phylogenetic structure of organisms can be created with clone libraries, as was done for FreshTrain. New sequencing technologies, such as the long reads produced by Nanopore (30) and PacBio (31, 32) instruments, promise even easier reference sequence generation in the future.

### Practical guidance for using TaxAss.

TaxAss includes detailed descriptions of its constituent scripts, including argument options and descriptions, so users are able to customize their analyses. The most important decision users make is the cutoff percent identity that determines which database is used to classify each OTU. If an OTU is above the cutoff (i.e., has a high percent identity to an ecosystem-specific reference sequence), then it will be classified with the ecosystem-specific database. When the cutoff is higher, fewer OTUs are classified using the ecosystem-specific database, and users run the risk of leaving some ecosystem-specific OTUs poorly classified or underclassified by the comprehensive database. When the cutoff is lower, more OTUs will be classified with the ecosystem-specific database, and users run the risk of overclassifications and misclassifications or of losing taxonomic richness due to underclassifications. Users can decide on a percent identity cutoff at the beginning and run only one classification, or they can run TaxAss with several cutoffs and generate versions of Fig. 5 to help guide their choice.

We found that a percent identity cutoff of 98 to 99 optimized classifications in our test data sets. The finding that most OTUs match their ecosystem-specific reference sequences with such a high percent identity suggests that the commonly chosen 97% sequence identity clustering is too coarse to observe fine-resolution dynamics. This is supported by previous findings that sequence identity-based OTUs can impose artificial delineations between organisms that affect results differently depending on the lineage (33) and that sequence identity-based OTUs can contain temporally discordant sequences (34). We recommend that users planning a taxonomy-centric analysis classify unique sequences after quality trimming and use fine-level taxonomic assignments to group their data instead of sequence identity cutoffs. The classification step will take longer with a larger number of unique sequences, but users will likely save computational time overall by not clustering. For users who also want to emphasize traditional OTU-based analyses, we recommend choosing a finer sequence identity-based OTU definition such as 98 or 99% to best leverage the fine-level classification provided by TaxAss and a detailed ecosystem-specific database. When OTUs have been clustered based on sequence identity, we recommend that users choose the same or lower percent identity cutoff in TaxAss to prevent an OTU's constituent sequences from falling on either side of the percent identity cutoff. We recommend choosing a percent identity cutoff using metrics similar to those recommended for unique sequences when finely resolved OTUs are defined with techniques such as DADA2 denoising (5) or minimum entropy decomposition (3).

### TaxAss informs ecological analyses.

Taxonomy-based analyses allow researchers to compare results across data sets. Leveraging an ecosystem-specific database for taxonomy assignment results in a high proportion of fine-resolution classifications, and grouping sequences based on these classifications is a data set-independent way to describe community composition. The resulting taxonomic terminology is consistent and comparable between analyses, and the finely resolved taxonomic groupings enable ecologically informed analyses. TaxAss can complement OTU-based analyses independent of the users’ chosen OTU definitions. Redefining OTUs to compare across data sets is computationally expensive and is not possible for data sets created with different amplification primers. When researchers use TaxAss to assign fine-level taxonomy to their data sets, colleagues can compare their results directly, without reanalysis and regardless of primer set. Additionally, taxonomic nomenclature can also bridge amplicon-based analyses and genomic analyses.

Leveraging ecosystem-specific databases for taxonomy assignment also improves researchers’ interpretations of individual data sets. Ecosystem-specific terminology is more meaningful because ecosystem-specific databases incorporate additional reference sequences, finer phylogenetic delineations, consistent nomenclature for uncultured organisms, and monophyletic structures. For example, the dominant lineage in freshwater is the FreshTrain’s actinobacterial lineage acI, which in SILVA is usually classified as family *Sporichthyaceae*. Although a classification exists for this organism in both databases, the SILVA family is much broader and also includes the separate FreshTrain lineages acSTL and acTH1. The FreshTrain’s finer-level phylogenetic information on these abundant freshwater *Actinobacteria* is based on manually curated alignments and ecological information, such as their occurrence in different lakes, and is supported by prior work suggesting the clades and tribes are ecologically and metabolically differentiated (15, 35, 36). The fine-resolution taxonomy assignments provided by TaxAss and an ecosystem-specific database allow researchers to link their amplicon data sets with known ecophysiological traits.

Ecosystem-specific phylogenies that are not fully incorporated into a comprehensive database are not straightforward to leverage for taxonomy assignment. The FreshTrain, for example, has diverged from Greengenes since it was created, and it has been used for taxonomy assignment with inconsistent and sometimes unreliable methods. TaxAss is a well-documented, open source, and rigorously tested workflow that avoids the pitfalls of using a small database: forcing incorrect classifications onto sequences and losing taxonomic richness by leaving unrepresented organisms unclassified. At the same time, TaxAss achieves the benefits of an ecosystem-specific database: more meaningful nomenclature, larger proportions of the data set classified, and finer-resolution classifications.

## MATERIALS AND METHODS

### How to use TaxAss.

TaxAss replaces only the taxonomy assignment step of users’ preferred amplicon data set processing pipeline such as mothur, qiime, or DADA2. TaxAss consists of a series of scripts using R, Python, bash, mothur, and BLAST that are run from the terminal command line singly or as a batch file. The input to TaxAss is a quality-controlled fasta file, and if users opt to run the optional percent identity cutoff metrics, a relative abundance table is also required. The output of TaxAss is the fasta file’s sequence IDs followed by their 7-level taxonomy assignments. Scripts, step-by-step instructions, and detailed explanations of script argument options are available online at https://github.com/McMahonLab/TaxAss.

### Percent identity recalculation.

The naive Bayesian algorithm used for taxonomy assignment (the Wang classifier) (16) can overclassify or misclassify OTUs when a close match does not exist in a small reference database. TaxAss uses the well-accepted Wang classifier, but avoids classification errors resulting from the effects of a small database by only classifying sequences for which a close reference exists. The National Center for Biotechnology Information’s Basic Local Alignment Search Tool (BLAST) (37) is utilized to split the amplicon data set into two groups prior to classification: one is classified with the ecosystem-specific database, the other with the comprehensive database.

blastn queries each OTU sequence against the ecosystem-specific database using the default megablast settings, which are optimized to find highly similar matches between sequences longer than 30 bp (24). However, BLAST returns the percent identity of the highest-scoring pair (the “pident”), which does not necessarily include the full length of the query OTU sequence. OTU sequences are highly similar; a single mismatch can change a classification, so mismatches at the ends of OTU sequences (in the “overhang”) that BLAST leaves out of the alignment must be included in the percent identity cutoff used for classification. Therefore, the BLAST pident is recalculated to a full-length percent identity with the following equation: percent identity = (pident × length)/{qlen + [length − (qend − qstart +1)]}, where “pident” is the percent identity returned by BLAST, “length” is the length of the alignment, “qlen” is the query length, “qend” is the query end, and “qstart” is the query start. All of these parameters are returned by BLAST output format 6, and detailed descriptions of what they are, the equation derivation, and an example alignment and calculation are included in Text S1.

The recalculation to full-length percent identity is conservative; all query nucleotides not included in the alignment (i.e., nucleotides in the “overhang”) are considered mismatches. This means that it would be possible to exclude an OTU from the ecosystem-specific classification when its true percent identity is above the cutoff due to matches in unaligned overhangs. An example of this situation is illustrated in Text S1. When the highest-scoring BLAST alignments contain matches on the overhangs, some of the lower-scoring alignments will be longer and therefore have a higher recalculated percent identity. To correct for this, TaxAss recalculates the percent identity of the top five BLAST hits and uses the best one for the cutoff decision. TaxAss also shows users the distribution of chosen hits, so that settings can be reevaluated if BLAST is not primarily returning hits that have the best recalculated percent identities.

### Cutoff choice.

OTUs with percent identities greater than or equal to the users’ specified cutoff are classified with the ecosystem-specific database using the Wang classifier as implemented by mothur. The remaining OTUs are classified with the comprehensive database, also using the Wang classifier. The choice of a percent identity cutoff is left to users so that they can balance their choices based on the structures of their data sets and their plans for analysis. If the percent identity cutoff is too low, dissimilar OTUs will be classified in the ecosystem-specific database and may be left unclassified or misclassified, but if the percent identity cutoff is too high, OTUs similar to the ecosystem-specific database will be classified by the comprehensive database and may end up underclassified.

Users have the option to choose a cutoff percent identity at the start, or they can classify their data sets with multiple cutoffs and TaxAss will provide metrics to guide their decisions. These metrics include versions of Fig. 5, which shows the cutoff choices that maximize the proportion of data set classified at different taxa levels.

As an additional optional check, users can also classify their data sets with only the comprehensive database and then compare the classifications. TaxAss provides metrics to check for coarse-resolution misclassifications. Phylum- and class-level classifications are more reliable when assigned by a large comprehensive database that includes more diversity, so if ecosystem-specific classifications at these coarse taxon levels disagree with the comprehensive database’s assignments, this suggests that the percent identity cutoff is too low. Only these coarse levels can be used to check for misclassifications because at finer taxonomic levels too many OTUs end up unclassified by the comprehensive database to compare assignments.

### Data availability and processing.

The freshwater tag data sets used in this paper are all publicly available on the National Center for Biotechnology Information’s (NCBI) Sequence Read Archive (SRA). The accession numbers are as follows: Lake Mendota, ERP016591 (34); Trout Bog, ERP016854 (22); Lake Michigan, SRP056973 (20); and Danube River, SRP045083 (21). The Lake Michigan and bog project accession numbers include additional sample types, so only the Lake Michigan and Trout Bog samples were used. The mouse gut data set is the full version of the example data used by mothur’s miSeq SOP, and is available on the mothur website (https://www.mothur.org/wiki/MiSeq_SOP) (23). The Marathonas Reservoir clone library data set is available from GenBank under accession no. GQ340065–GQ340365 (19). The Marathonas Reservoir taxonomy determined by manual alignment to the FreshTrain is available from https://www.github.com/McMahonLab/TaxAss.

The taxonomy databases used in this article are also publicly available. The Freshwater Training Set (FreshTrain) version used was FreshTrain30Apr2018SILVAv132 (15), which includes 1,318 freshwater heterotrophic bacterial references and is available from https://www.github.com/McMahonLab/TaxAss. The SILVA database version used was version SSU 132 NR 99 (https://www.arb-silva.de) (8), which includes 213,119 bacterial and archaeal reference sequences clustered to 99% identity to avoid repeat references. A mothur-formatted version of this database obtained from https://www.mothur.org/wiki/Silva_reference_files was used for all analyses (accessed January 2018). Further details on download, versions, and formatting can be found in Text S2 in the supplemental material and in the detailed directions provided at https://www.github.com/McMahonLab/TaxAss.

10.1128/mSphere.00327-18.2 TEXT S2  Batch files for data processing. This document includes directions and commands for reproducing all data processing in this paper. It pairs with a zip file hosted on the TaxAss github repository, which includes the folder structure and scripts used to download and process all tag data sets used in this article. The folders contain batch scripts that download each data set, batch scripts that quality control each data set, and batch scripts that perform TaxAss on each data set, along with the versions of TaxAss scripts used in this article. Download TEXT S2, PDF file, 0.2 MB. Copyright © 2018 Rohwer et al. 2018 Rohwer et al. This content is distributed under the terms of the Creative Commons Attribution 4.0 International license.

Quality control of tag data set fastq files was performed according to mothur's MiSeq SOP (23, accessed September 2017) through the chimera checking step with mothur version 1.39.5 (17). The resulting unique sequences were defined as OTUs for all further analyses. The single-end sequencing data sets (Lake Mendota and Trout Bog) were also preprocessed with vsearch version 2.3.4_osx_x86_64 (38) to trim to uniform lengths and remove low-quality sequences with >0.5 expected errors. During TaxAss, the percent identity cutoff used for all data sets was 98, and the Wang classifier’s bootstrap confidence was set at 80% for all classifications. Batch files that reproduce all download, quality control, and TaxAss processing for each data set are available in Text S2.

Manual alignment of the full-length Marathonas Reservoir clone library sequences to the FreshTrain database was performed using the program ARB (39). Chimeras were manually identified and removed from the analysis, and sequences without FreshTrain nomenclature were labeled unclassified. Tags were simulated by trimming full-length sequences to common primer regions with mothur version 1.39.5 (17). The primers used were V4 (515F, GTGCCAGCMGCCGCGGTAA; 806R, GGACTACHVGGGTWTCTAAT) (40), V4-V5 (515FB, GTGYCAGCMGCCGCGGTAA; 926R, CCGYCAATTYMTTTRAGTTT) (41), and V3-V4 (341F, CCTACGGGNGGCWGCAG; 805R, GACTACHVGGGTATCTAATCC) (42). A list of the processing commands used to trim sequences to the primer regions and classify them is available in Text S3 in the supplemental material.

10.1128/mSphere.00327-18.3 TEXT S3  Tag simulation processing commands. This document explains in more detail the TaxAss validation using tags simulated from the full-length Marathonas clone library data. It also includes all commands necessary to reproduce the simulation. Download TEXT S3, PDF file, 0.2 MB. Copyright © 2018 Rohwer et al. 2018 Rohwer et al. This content is distributed under the terms of the Creative Commons Attribution 4.0 International license.
