# Supplementary material for: TaxAss: Leveraging a Custom Freshwater Database Achieves Fine-Scale Taxonomic Resolution
Source: mSphere. 2018 Sep 5;3(5):e00327-18. doi: 10.1128/mSphere.00327-18 (PMC6126143; doi:10.1128/mSphere.00327-18)
Supplement: TABLE S1 [file sph004182626st1.pdf]

	Simulated V4 Tags			Simulated V4-V5 Tags			Simulated V3-V4 Tags		
	Lineage	Clade	Tribe	Lineage	Clade	Tribe	Lineage	Clade	Tribe
Overclassifications	0	0	3	0	1	3	0	0	4
Misclassifications	0	2	2	0	2	3	0	0	2
Incorrectly in FreshTrain	5	5	5	1	1	1	2	2	2
Incorrectly in SILVA	62	62	62	62	62	62	58	58	58
Underclassifications	0	4	10	0	5	7	0	6	5
Correctly in SILVA	110	110	110	114	114	114	113	113	113
Correct Unclassifications	0	14	24	0	13	20	0	15	22
Correct Classifications	108	88	69	108	87	75	112	91	79
