# Supplementary figures and images for: TaxAss: Leveraging a Custom Freshwater Database Achieves Fine-Scale Taxonomic Resolution
Source: mSphere. 2018 Sep 5;3(5):e00327-18. doi: 10.1128/mSphere.00327-18 (PMC6126143; doi:10.1128/mSphere.00327-18)

A

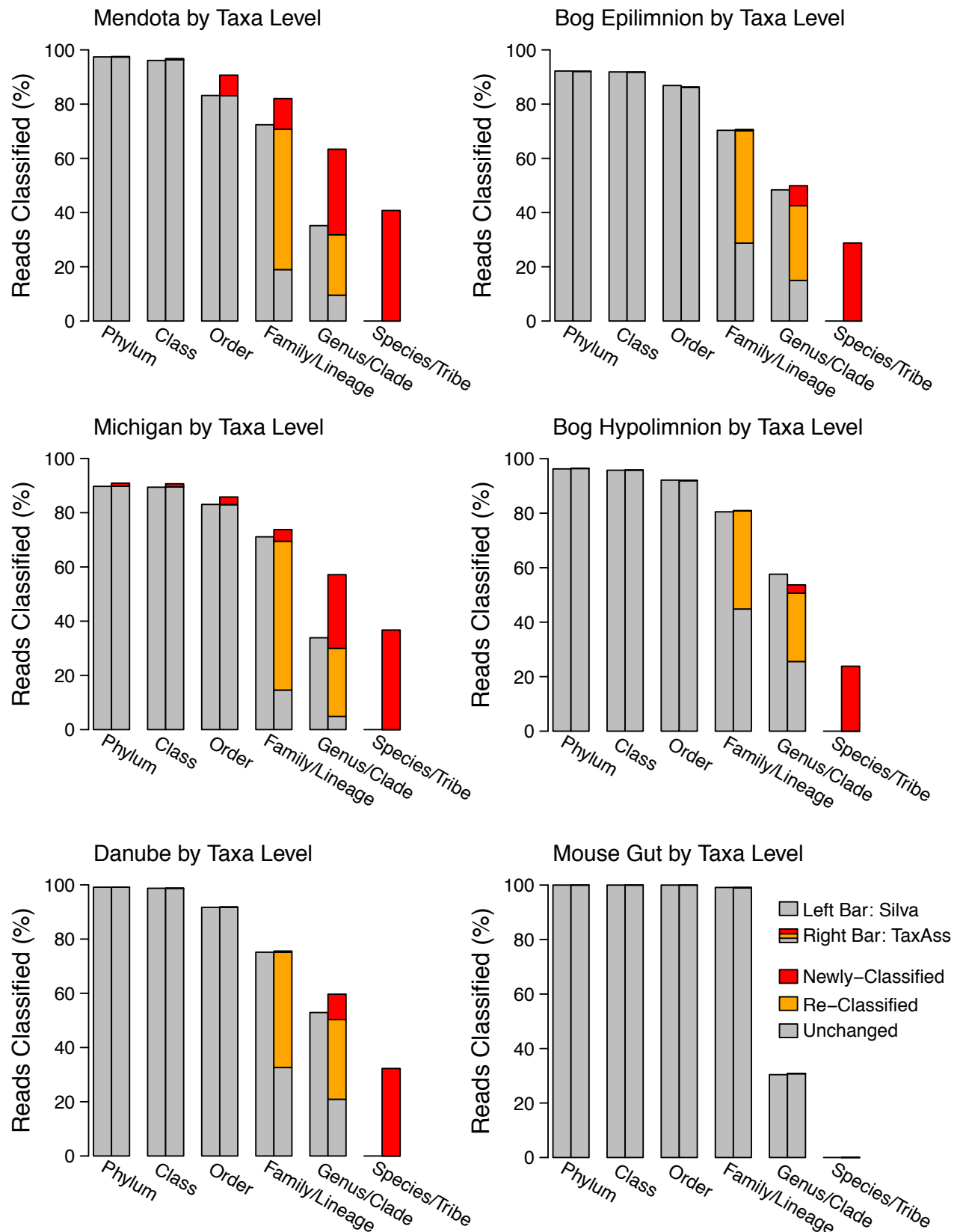

B

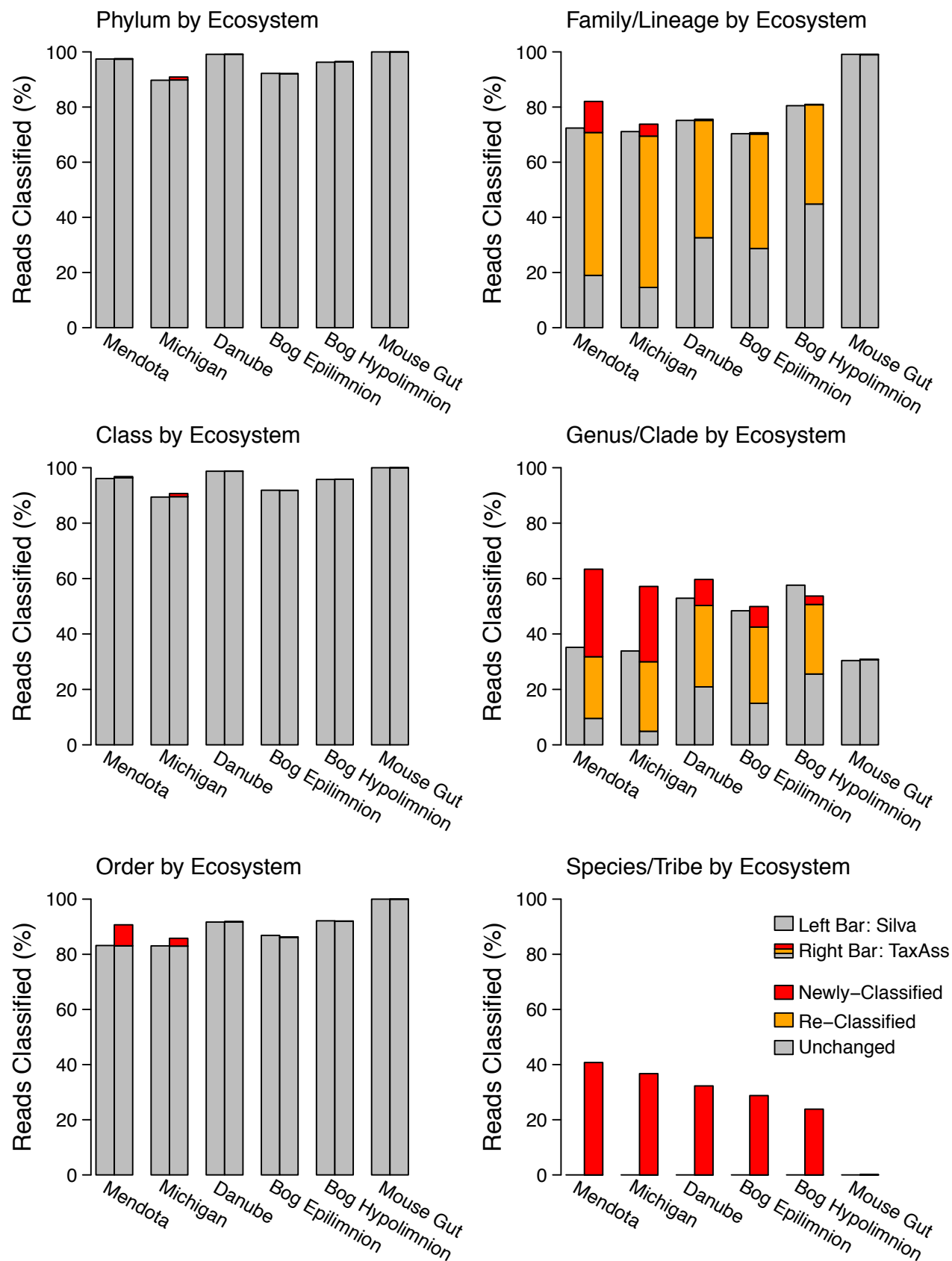

Supplement: FIG S1 [file sph004182626sf1.pdf]

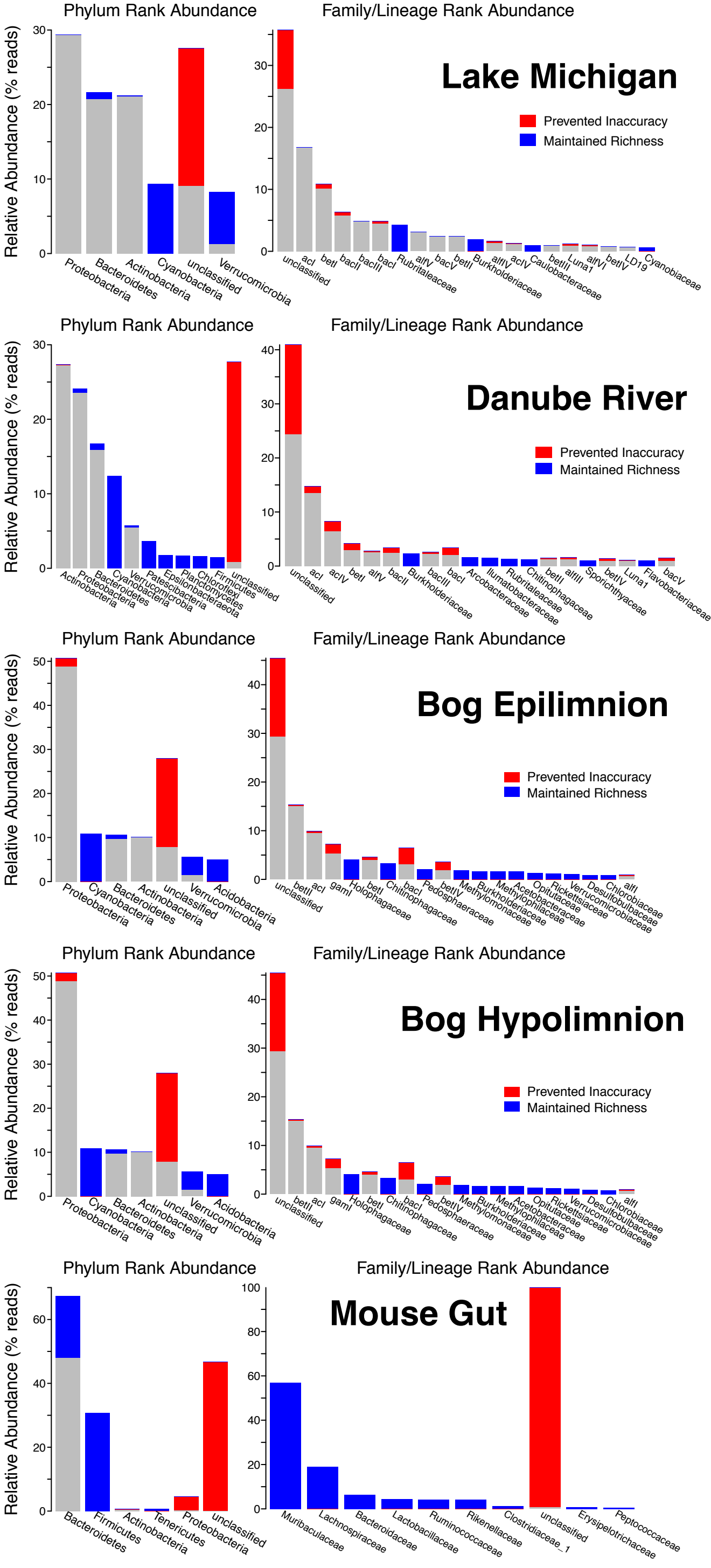

Supplement: FIG S2 [file sph004182626sf2.pdf]

# Cyanobacteria Percent Identity Recalculations

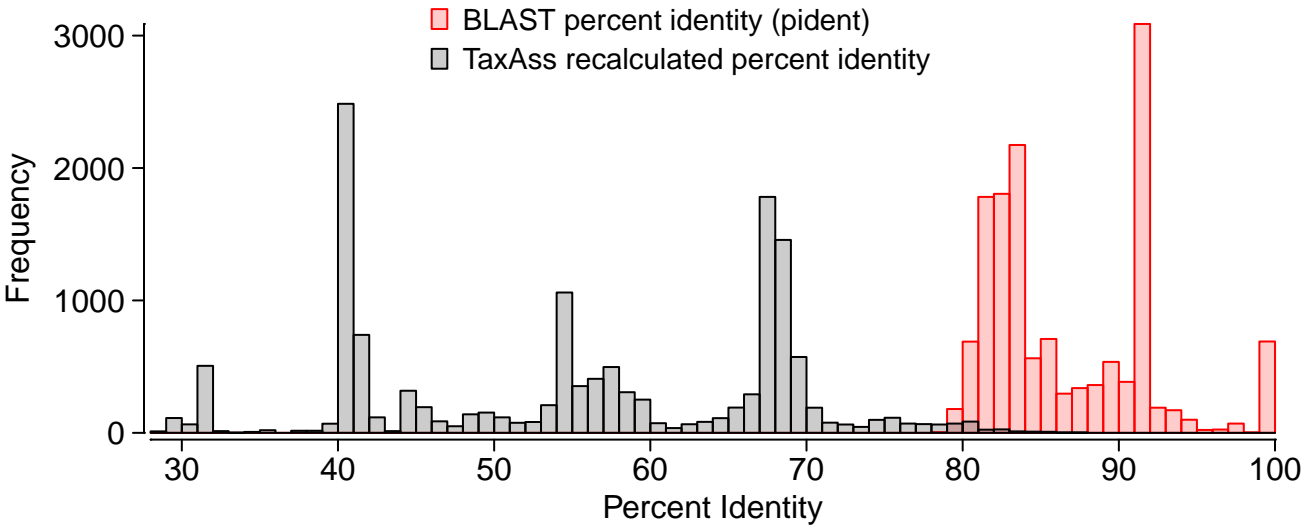

Supplement: FIG S3 [file sph004182626sf3.pdf]
