# Supplementary material for: TaxAss: Leveraging a Custom Freshwater Database Achieves Fine-Scale Taxonomic Resolution
Source: mSphere. 2018 Sep 5;3(5):e00327-18. doi: 10.1128/mSphere.00327-18 (PMC6126143; doi:10.1128/mSphere.00327-18)
Supplement: TABLE S2 [file sph004182626st2.pdf]

		Percent Classified[a]			Taxonomic Richness[b]		
		Silva	TaxAss	FreshTrain	Silva	TaxAss	FreshTrain
Mendota	Phylum	97	98	85	63	63	6
	Class	96	97	84	160	162	10
	Order	83	91	76	387	388	31
	Family/Lineage	72	82	69	700	742	57
	Genus/Clade	35	63	56	1468	1529	94
	Species/Tribe	0	41	41	1468	1579	147
Michigan	Phylum	90	91	72	49	48	6
	Class	89	91	71	136	135	10
	Order	83	86	67	340	339	31
	Family/Lineage	71	74	64	670	712	57
	Genus/Clade	34	57	55	1829	1908	93
	Species/Tribe	0	37	39	1829	1954	143
Danube	Phylum	99	99	72	71	71	5
	Class	99	99	71	194	192	9
	Order	92	92	65	500	496	30
	Family/Lineage	75	76	59	891	924	56
	Genus/Clade	53	60	46	2280	2322	92
	Species/Tribe	0	32	36	2280	2374	148
Bog Epilimnion	Phylum	92	92	72	60	57	5
	Class	92	92	66	139	136	9
	Order	87	86	57	319	312	30
	Family/Lineage	70	71	55	554	585	53
	Genus/Clade	48	50	42	1112	1147	82
	Species/Tribe	0	29	32	1112	1186	124
Bog Hypolimnion	Phylum	96	96	72	60	58	5
	Class	96	96	61	145	138	9
	Order	92	92	50	324	319	30
	Family/Lineage	80	81	49	568	596	54
	Genus/Clade	58	54	35	1062	1086	83
	Species/Tribe	0	24	25	1062	1125	126
Mouse Gut	Phylum	100	100	53	18	18	5
	Class	100	100	49	28	28	9
	Order	100	100	1	60	60	22
	Family/Lineage	99	99	0	102	115	35
	Genus/Clade	30	31	0	180	188	41
	Species/Tribe	0	0	0	180	196	51
