# Supplementary material for: TaxAss: Leveraging a Custom Freshwater Database Achieves Fine-Scale Taxonomic Resolution
Source: mSphere. 2018 Sep 5;3(5):e00327-18. doi: 10.1128/mSphere.00327-18 (PMC6126143; doi:10.1128/mSphere.00327-18)
Supplement: TEXT S1 [file sph004182626s1.pdf]

BLAST Terminology Definitions

To understand the conversion of a BLAST pident to the full length percent identity used as a cutoff in TaxAss, first you must understand the BLAST terminology. Below are some basic BLAST terms:

Query	The sequence you input into BLAST<br>(here, an OTU sequence)
Subject	The reference sequence from your BLAST database that matched the query<br>(here, a full length 16S sequence from the taxonomy database)
HSP	"High Scoring Pair" This is the matching section of DNA sequences that BLAST returns. It is scored based on many criteria, with different weights given to different types of mismatches/gaps and to the length of the match.

BLAST can return many different statistics, but below are the ones used to calculate the workflow's full length percent identity cutoff. Note that some of these statistics, such as "length," have a different meaning in different output formats.

BLAST output format 6 term:	Stands for:	Description:	References which sequence?	Includes gaps and mismatches?
length	"alignment length"	number of nucleotides in the HSP	HSP	YES
qlen	"query length"	number of nucleotides in the query	Query	NO
qstart	"query start"	first query nucleotide included in the HSP	Query	NO
qend	"query end"	last query nucleotide included in the HSP	Query	NO
pident	"percent identity"	percent of nucleotides in the HSP that match	HSP	YES

In order to calculate the full length percent identity used as a cutoff in TaxAss, several new statistics that BLAST does not provide are calculated. Descriptions of these statistics are below:

Workflow term:	Description:	References which sequence?	Includes gaps and mismatches?
# matches	number of matching nucleotides in the HSP	HSP	NO
query gaps	number of gaps in the query sequence side of the alignment	HSP	YES
query nucleotides in HSP	number of nucleotides in the query sequence side of the alignment	Query	NO
full length	query length (qlen) plus any query gaps	Query & HSP	YES
full length pident	# matches / full length * 100%.	Query & HSP	YES

Note that the full length percent identity is a conservative average nucleotide identity (ANI) because all nucleotides not included in the HSP (in BLAST terms, "overhang") are counted as mismatches.

Equation Derivation

Below is the derivation of the equation that calculates the full length percent identity used as a cutoff in TaxAss (BLAST terms in blue, TaxAss terms in black):

$$\text{full length pident} = \frac{\text{\# matches}}{\text{full length}} \times 100\%$$

$$\left\{ \begin{aligned} &\text{pident} = \frac{\text{\# matches}}{\text{length}} \times 100\% \\ &\text{full length pident} = \frac{\text{\# matches}}{\text{length}} \times \frac{\text{length}}{1} \times \frac{1}{\text{full length}} \times 100\% \end{aligned} \right.$$

$$\text{full length pident} = \frac{\text{pident}}{1} \times \frac{\text{length}}{1} \times \frac{1}{\text{full length}}$$

$$\left\{ \begin{aligned} &\text{full length} = \text{qlen} + \text{query gaps} \\ &\text{query gaps} = \text{length} - \text{query nucleotides in HSP} \\ &\text{query nucleotides in HSP} = \text{qend} - \text{qstart} + 1 \\ &\text{full length} = \text{qlen} + (\text{length} - (\text{qend} - \text{qstart} + 1)) \end{aligned} \right.$$

$$\text{full length pident} = \frac{\text{pident}}{1} \times \frac{\text{length}}{1} \times \frac{1}{\text{qlen} + (\text{length} - (\text{qend} - \text{qstart} + 1))}$$

$$\boxed{\text{full length pident} = \frac{\text{\# matches}}{\text{full length}} \times 100\% = \frac{\text{pident} \times \text{length}}{\text{qlen} + (\text{length} - (\text{qend} - \text{qstart} + 1))}}$$

Example Calculation

The following example OTU and reference sequence were run through TaxAss. This example includes all possible gap and mismatch types:

```
>otu
TACGAAAGGGGCAAGCGTTATCCGGAATTATTGGGCGTAAAGAGCTCGTAGGTCGTTTGTCGCGTCGATTGTGAAAATCTGAGGCTCAACCTCAGC

>reference
AGAGTTTGATCATGGCTCAGGACGAACGCTGGCGGCGTGCTTTATACATGCAAGTCGAACGATGAAGTTCCTTCGGGAATGGATTAGTGGCGAACGGGTGAGGAACACGTGAGAAACCTGCCTTTCATTCTGGGA
TAACACCGGGAAACCGGTGCTAATACCGGATACTCCATCTTGGCGGCATCGCCGAGCTGGGAAAAAAATTTTGATGAAAGATGGTCTCGCGGCCTATCAGCTAGTTGGTGAGGTAAAGGCTCACCAAGGCGACGAC
GGGTAGCCGGCCTGAGAGGGCGACCGGCCACACTGGGACTGAGACACGGCCCAGACTCCTACGGGAGGCAGCAGTAGGGAATATTGGGCAATGGGCGAAAGCCTGACCCAGCGACGCCGCGTGAGGGATGAAGGT
CTTCGGATTGTAAACCTCTTTCAGTAGGGAAGAAGCGAAAGTGACGGTACCTAAAGAAGAAGCACCGGCTAACTATGTGCCAGCAGCCGCGGTAATACATAGGGTGCAAGCGTTGTCCGGAATTATTGGGCGTAA
AGAGCTCGTAGGTCGTTTGTCGCGTCGATTGTGAAATCTGAGGCTCAACCTCAGACCTGCAGTCGATACGGGCAAACTAGAGTGTGGTAGGGGAGACTGGAATTCCTGGTGTAGCGGTGGAATGCGCAGATATCA
GGAGGAACACCAATGGCGAAGGCAGGTCTCTGGGCCATAACTGACACTGAGGAGCGAAAGTGCGGGGAGCGAACAGGATTAGATACCCTGGTAGTCCGCACCGTAAACGGTGGGCACTAGTTGTGGGAACCTTCC
ACGGTTTCCGCGACGCAGCAAACGCATTAAGTGCCCCGCCTGGGGAGTACGATCGCAAGATTAAAACTCAAAGGAATTGACGGGGCCCCGCACAAGCAGCGGAGCATGCGGCTTAATTCGACGCAACGCGAAGAA
CCTTACCAAGGCTTGACATATAGCGAAAAGTGGCAGAGATGTCATGTCCGCAAGGGCGCTATACAGGTGGTGCATGGTTGTCGTCAGCTCGTGTCGTGAGATGTTGGGTTAAGTCCCGCAACGAGCGCAACCCTC
GTTCTGTGTTGCCAGCATTTAGTTGGGGACTCACAGGAGACTGCCGGGGTCAACTCGGAGGAAGGTGGGGATGACGTCAAATCATCATGCCCCTTATGTCTTGGGCTGCACGCATGCTACAATGGCTGGTACAAA
GGGCTGCAATACTGCAAAGTGGAGCGAATCCCAAAAAGCCAGTCTCAGTTCGGATTGGGGTCTGCAACTCGACCCCATGAAGTCGGAGTTGCTAGTAATCGTAGATCAGCAACGCTACGGTGAATACGTTCCCGG
GGCTTGTACACACCGCCCGTCACATCACGAAAGTCGGTAACACCCGAAGTCAGTGGCCCAACCGTAAGGGGGGAGCTGCCGAAGGTGGGATCGGTGATTGGGATGAAGTCGTAACAAGGTA
```

Here is a display of the alignment modified from BLAST output format 3 to show the entire query sequence. The aligned section is bold, the unaligned sections are grey, and mismatches and gaps are red. Matches that are conservatively called mismatches by the workflow are in green.

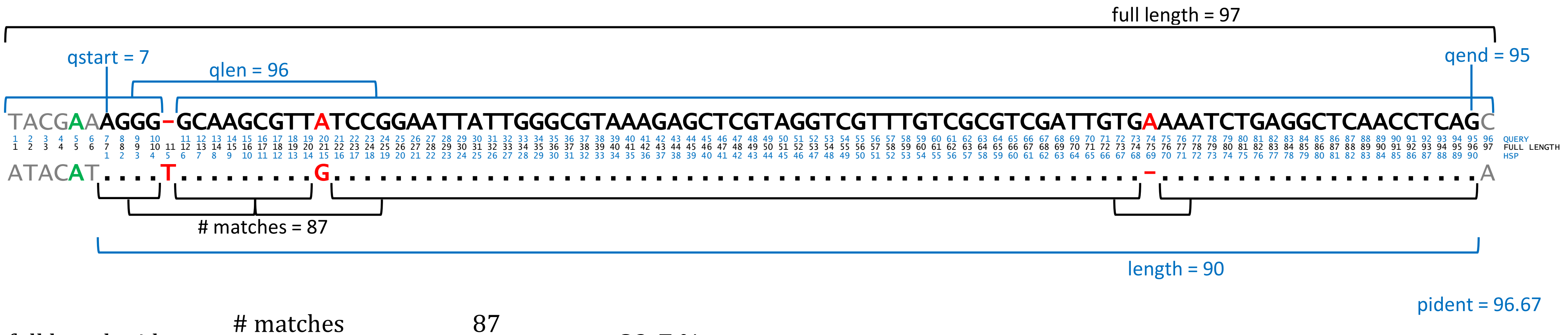

$$\text{full length pident} = \frac{\#\text{ matches}}{\text{full length}} \times 100\% = \frac{87}{97} \times 100\% = \mathbf{89.7}\ \%$$

$$\text{full length pident} = \frac{\text{pident} \times \text{length}}{\text{qlen} + (\text{length} - (\text{qend} - \text{qstart} + 1))} = \frac{96.67 \times 90}{96 + (90 - (95 - 7 + 1))} = \mathbf{89.7}\ \%$$

Note that this calculation is conservative; the true percent identity should include the green match and be $\frac{\mathbf{88}}{\mathbf{97}} = \mathbf{90.7}\ \%$. The diagnostic plot in step 6 helps you identify if this is a problem, but with recommended percent identity cutoffs in the range of 97-99% an overhang can only be 1-2 nucleotides long . Likely if an overhang is long enough for matches to occure on it, as above, the sequence would not make the cutoff anyway.
