# Supplementary material for: TaxAss: Leveraging a Custom Freshwater Database Achieves Fine-Scale Taxonomic Resolution
Source: mSphere. 2018 Sep 5;3(5):e00327-18. doi: 10.1128/mSphere.00327-18 (PMC6126143; doi:10.1128/mSphere.00327-18)
Supplement: TEXT S2 [file sph004182626s2.pdf]

README-TaxAss-BatchFiles

Robin Rohwer

March 13, 2018

Reproduce Data Processing in TaxAss Paper

A better-formatted html version of these directions are in the github repo `figure-scripts` folder. That's also where the zip file containing all the folder structures and batch scripts are. msphere doesn't accept html or zip file formats, so that's why a crappy pdf is the placeholder in the supplemental info.

Download versions of programs and databases used in this paper:

Program	Version	Download From
mothur	v.1.39.5	mothur.org
SraToolkit	2.7.0-mac64	ncbi.nlm.nih.gov
BLAST	2.2.31+	ncbi.nlm.nih.gov
vsearch	2.4.3-macos-x86_64	github.com/torognes/vsearch
TaxAss	SHA 8797aa9	github.com/McMahonLab/TaxAss/tax-scripts (And included in these batch files)

Database	Version	File Name	Download From
FreshTrain	18Aug2016	FreshTrain18Aug2016.zip	github.com/McMahonLab/TaxAss/FreshTrain-files
Silva NR99	V132	Silva.nr_v132	https://mothur.org/wiki/Silva_reference_files
Silva Seed	v128	silva.seed_v128.align	https://mothur.org/wiki/Silva_reference_files

Add these programs to your path after installing:

- mothur
- SraToolkit
- BLAST (probably automatically installs to path)

The TaxAss tax-scripts will already be in each ecosystem folder here. Otherwise you would add them to your working directory (not on the path).

Download the reference databases into the existing folder called ReferenceDatabases:

- FreshTrain
- Silva
- Silva seed

Unzip the files, and move them so that they are not nested within other folders after expanding. Do not change any names. This is what the contents of `ReferenceDatabases` should look like:

```
ls ReferenceDatabases/

# FreshTrain25Jan2018SILVAv132.fasta
# FreshTrain25Jan2018SILVAv132.taxonomy
# README-FreshTrain25Jan2018SILVAv132.txt
# README.txt
# silva.nr_v132.align
# silva.nr_v132.tax
# silva.seed_v128.align
```

Re-format the reference databases used by TaxAss:

Simply re-name the ecosystem-specific FreshTrain:

```
mv FreshTrain25Jan2018SILVAv132.fasta custom.fasta
mv FreshTrain25Jan2018SILVAv132.taxonomy custom.taxonomy
```

The silva database does not *need* to be reformatted, but right now it is aligned and it does not *need* to be aligned. By un-aligning it will save ~10 GB of space:

```
./un-align_silva.sh silva.nr_v132.align general.fasta silva.nr_v132.tax general.taxonomy
```

Navegate into the main TaxAss-BatchFiles folder within the terminal.

These notes assume starting in that folder as the present working directory at the beginning of each of the following "Process . . . " steps.

This main direcory contains lake-specific folders for processing each dataset, in addition to the Reference-Databases folder you downloaded files into yourself and the `tax-scripts` folder taken from the TaxAss github repository.

```
ls

# Danube/
# Mendota/
# Michigan/
# MouseGut/
# ReferenceDatabases/
# TroutBogEpi/
# TroutBogHypo/
# tax-scripts/
```

Each lake-specific folder contains separate folders for different processing steps. For example:

```
ls Mendota/

# QC-Mendota/
# TaxAss-Mendota/
```

Notes on these batch files:

- QC downloads raw fastq files and converts them to fasta files using standard QC settings.

- TaxAss runs the full TaxAss workflow, including optional steps to identify a good percent identity cutoff.
- These batch files are "all inclusive" because they're just meant to provide reproducible results. If you're running TaxAss on your own data and want to run as a batch file, you should adjust the provided batch files within the tax-scripts folder with names `RunSteps_*.sh`.
- Note that script annotations include more details, especially the QC scripts which are not included as part of the TaxAss workflow.

- Sending the terminal output to a text file allows you to check for errors later. Deleting the 1000's of lines of numbers mothur prints while processing ("mothur barf") makes the output readable.

Process Lake Mendota!

```
cd Mendota/QC-Mendota/
./qc_mendota.sh > terminal_output.txt
./deletemothurbarf.sh terminal_output.txt > terminal_output_qc.txt ; rm terminal_output.txt

cd ../TaxAss-Mendota/
./taxass_mendota.sh
```

Process Trout Bog Epilimnion!

```
cd TroutBogEpi/QC-TroutBogEpi/
./qc_bog_epi.sh > terminal_output.txt
./deletemothurbarf.sh terminal_output.txt > terminal_output_qc.txt ; rm terminal_output.txt

cd ../TaxAss-TroutBogEpi/
./taxass_bog_epi.sh
```

Process Trout Bog Hypolimnion!

```
cd TroutBogHypo/QC-TroutBogHypo/
./qc_bog_hypo.sh > terminal_output.txt
./deletemothurbarf.sh terminal_output.txt > terminal_output_qc.txt ; rm terminal_output.txt

cd ../TaxAss-TroutBogHypo/
./taxass_bog_hypo.sh
```

Process Lake Michigan!

```
cd Michigan/QC-Michigan/
./qc_michigan.sh > terminal_output.txt
./deletemothurbarf.sh terminal_output.txt > terminal_output_qc.txt ; rm terminal_output.txt

cd ../TaxAss-Michigan/
./taxass_michigan.sh
```

Process Danube River!

```
cd Danube/QC-Danube/
./qc_danube.sh > terminal_output.txt
./deletemothurbarf.sh terminal_output.txt > terminal_output_qc.txt ; rm terminal_output.txt

cd ../TaxAss-Danube/
./taxass_danube.sh
```

Process Mouse Gut!

This data is the full version of the example used in mothur's MiSeq SOP (aka the "stability" study for those of you who've also memorized it). Go to the MiSeq SOP: https://www.mothur.org/wiki/MiSeq_SOP and scroll down to the "Logistics" section. Then click the "full dataset" link and it will open a download window. Download into the `TaxAss-BatchFiles/MouseGut/QC-MouseGut/` folder.

```
cd MouseGut/QC-MouseGut
# download dataset from mothur's website into this folder
tar -xvf StabilityNoMetaG.tar
rm Mock* # delete mock community samples- not real mousegut data
gunzip *.gz
rm StabilityNoMetaG.tar # delete large file
./qc_mousegut.sh > terminal_output.txt
./deletemothurbarf.sh terminal_output.txt > terminal_output_qc.txt ; rm terminal_output.txt

cd ../TaxAss-MouseGut/
./taxass_mousegut.sh
```

Now you're all done replicating the data processing used in the TaxAss manuscript. Go use TaxAss on your own datasets!!!
