# Supplementary material for: TaxAss: Leveraging a Custom Freshwater Database Achieves Fine-Scale Taxonomic Resolution
Source: mSphere. 2018 Sep 5;3(5):e00327-18. doi: 10.1128/mSphere.00327-18 (PMC6126143; doi:10.1128/mSphere.00327-18)
Supplement: TEXT S3 [file sph004182626s3.pdf]

README_marathonas_validation

Robin Rohwer

1/22/2018

Reproduce TaxAss validation using full-length 16S assignments

This readme walks you through the data processing to reproduce "simulated tag data" from full-length 16S sequences. This was used to validate TaxAss.

The process:

- Start with full-length 16S sequences from Marathonas reservoir in Greece.
  - These do not already exist in the FreshTrain database.
- Align the full-length sequences to the FreshTrain manually using Arb.
  - The result of this is zipped in this folder. Likely chimeras were removed.
- Trim the full-length Marathonas sequences to a primer region of choice.
  - That's where this Readme picks up.
- Assign taxonomy to the short primer regions using TaxAss.
  - When I say "tax!", you say "ass!"
- Compare the TaxAss assignment results to the "true" classifications known from the full-length alignments.

Unzip and add TaxAss tax-scripts

Start with `arb-scripts/Marathonas_test/` as your present working directory.

Add the tax-scripts and taxonomy database files to the `add-tax-scripts-and-databases` folder:

```
# all the commands had to get wrapped because msphere doesn't accept html documents.
# you can get this better-formatted in the TaxAss repo.
cp ../../tax-scripts/*  add-tax-scripts-and-databases
# unzip your FreshTrain reference (using SILVA v132 for example:)
cp ../../FreshTrain-files/FreshTrain30Apr2018SILVAv132/FreshTrain30Apr2018SILVAv132.fasta \
add-tax-scripts-and-databases/custom.fasta
cp ../../FreshTrain-files/FreshTrain30Apr2018SILVAv132/FreshTrain30Apr2018SILVAv132.taxonomy \
add-tax-scripts-and-databases/custom.taxonomy
# add your comprehensive database (formatted according to TaxAss step 0,
# named general.fasta and general.taxonomy)
```

Trim full-length sequences to different V regions

Trim the Marathonas full-length 16S sequences to the variable regions amplified by primers in tag data. Do this for several commonly used primer sets that amplify regions of differing lengths. Put each V-region analysis in a different folder.

This uses the shell script `trim_full-length_to_primer_region.sh` and requires the program mothur to be on your computer's path. The syntax to call the script is:

```
./trim_full-length_to_primer_region.sh primers.oligos aligned-fasta-without-filetype \
name-for-trimmed.fasta
```

note: this is set to correct for a bug in mothur v.1.39.5, so script should be adjusted if a new verison fixes bug (says where in script)

V4 region

```
mkdir v4_mara
# add all the scripts and reference files
cp add-tax-scripts-and-databases/* v4_mara/
cp oligo-files/v4_515F-806R.oligos v4_mara/
cp oligo-files/ecoli_16S.fasta v4_mara/
cp trim_full-length_to_primer_region.sh v4_mara/
# add all the marathonas data files
cp mara_aligned.fasta v4_mara/
cp mara_dummy.abund v4_mara/otus.abund
# trim the fasta file to primer region
cd v4_mara
./trim_full-length_to_primer_region.sh v4_515F-806R.oligos mara_aligned otus.fasta
cd ../
```

V4-V5 region

```
mkdir v4v5_mara
# add all the scripts and reference files
cp add-tax-scripts-and-databases/* v4v5_mara/
cp oligo-files/v4v5_515FB-926R.oligos v4v5_mara/
cp oligo-files/ecoli_16S.fasta v4v5_mara/
cp trim_full-length_to_primer_region.sh v4v5_mara/
# add all the marathonas data files
cp mara_aligned.fasta v4v5_mara/
cp mara_dummy.abund v4v5_mara/otus.abund
# trim the fasta file to primer region
cd v4v5_mara
./trim_full-length_to_primer_region.sh v4v5_515FB-926R.oligos mara_aligned otus.fasta
cd ../
```

V3-V4 region

```
mkdir v3v4_mara
# add all the scripts and reference files
cp add-tax-scripts-and-databases/* v3v4_mara/
cp oligo-files/v3v4_341F-805R.oligos v3v4_mara/
cp oligo-files/ecoli_16S.fasta v3v4_mara/
cp trim_full-length_to_primer_region.sh v3v4_mara/
# add all the marathonas data files
cp mara_aligned.fasta v3v4_mara/
cp mara_dummy.abund v3v4_mara/otus.abund
# trim the fasta file to primer region
cd v3v4_mara
./trim_full-length_to_primer_region.sh v3v4_341F-805R.oligos mara_aligned otus.fasta
cd ../
```

TaxAss the simulated tag data

Run TaxAss on each simulated tag dataset.

V4 region

```
cd v4_mara
./RunSteps_1-14.sh > termout.txt
./deletemothurbarf.sh termout.txt > terminal_output_1-14.txt
./RunStep_15.sh > termout.txt
./deletemothurbarf.sh termout.txt > terminal_output_15.txt
rm termout.txt
# don't ./RunStep_16.sh, need some intermediate files for analysis
cd ../
```

V4-V5 region

```
cd v4v5_mara
./RunSteps_1-14.sh > termout.txt
./deletemothurbarf.sh termout.txt > terminal_output_1-14.txt
./RunStep_15.sh > termout.txt
./deletemothurbarf.sh termout.txt > terminal_output_15.txt
rm termout.txt
# don't ./RunStep_16.sh, need some intermediate files for analysis
cd ../
```

V3-V4 region

```
cd v3v4_mara
./RunSteps_1-14.sh > termout.txt
./deletemothurbarf.sh termout.txt > terminal_output_1-14.txt
./RunStep_15.sh > termout.txt
./deletemothurbarf.sh termout.txt > terminal_output_15.txt
rm termout.txt
# don't ./RunStep_16.sh, need some intermediate files for analysis
cd ../
```

Compare TaxAss names to full-length "true" names

Use script `compare_full-length_to_taxass_results.R` which can be called from the terminal with this syntax:

```
Rscript compare_full-length_to_taxass_results.R file.arb.tax file.v4.tax file.v4.ids folder.v4
```

argument	description
file.arb.tax	`mara.taxonomy`, the "true" taxonomy determined by aligning the full-length sequence
file.v4.tax	`otus.taxonomy`, the taxonomy assignmed to simulated tags by TaxAss
file.v4.ids	`ids.above.98`, an intermediate file made by TaxAss that lists the seqIDs above the percent identity cutoff.Note: this is the one that gets deleted if you `RunStep_16.sh`
folder.v4	a (pre-existing) folder that you want to save the results in

V4 region

```
cd v4_mara
mkdir v4_results
Rscript ../compare_full-length_to_taxass_results.R ../mara.silva132.taxonomy \
otus.98.80.80.taxonomy ids.above.98 v4_results
cd ../
```

V4-V5 region

```
cd v4v5_mara
mkdir v4v5_results
Rscript ../compare_full-length_to_taxass_results.R ../mara.silva132.taxonomy \
otus.98.80.80.taxonomy ids.above.98 v4v5_results
cd ../
```

V3-V4 region

```
cd v3v4_mara
mkdir v3v4_results
Rscript ../compare_full-length_to_taxass_results.R ../mara.silva132.taxonomy \
otus.98.80.80.taxonomy ids.above.98 v3v4_results
cd ../
```
